# Aortic perforation following percutaneous coronary intervention in a patient with permanent pacemaker: a case report

**DOI:** 10.1093/ehjcr/ytaf298

**Published:** 2025-06-24

**Authors:** Toe Wai Wai Naing, Xiaofang Chen, Yan Wang, Ye Cheng

**Affiliations:** Department of Cardiology, The Xiamen Cardiovascular Hospital of Xiamen University, Fujian 361000, China; Department of Cardiology, The Xiamen Cardiovascular Hospital of Xiamen University, Fujian 361000, China; Department of Cardiology, The Xiamen Cardiovascular Hospital of Xiamen University, Fujian 361000, China; Department of Cardiology, The Xiamen Cardiovascular Hospital of Xiamen University, Fujian 361000, China

**Keywords:** Cardiac tamponade, Aortic perforation, Percutaneous coronary intervention, Permanent pacemaker, Case report

## Abstract

**Background:**

Lead perforation is an uncommon complication of permanent pacemaker (PPM) implantation, occurring in 0.1–0.8% of cases (1). Aortic perforation caused by a pacing lead is rare and has been described only in case reports.

**Case summary:**

A 70-year-old Chinese man with a medical history of diabetes mellitus and hypertension presented to the hospital with intermittent chest pain of 3 weeks duration. He was diagnosed with chronic coronary syndrome and underwent percutaneous coronary intervention (PCI) to left anterior descending artery. Two days after post-PCI, he developed shock due to cardiac tamponade. A repeat angiogram was unremarkable; however, during thoracotomy, the right atrial pacing lead of a dual-chamber pacemaker, implanted 6 weeks previously for complete heart block, was found to have perforated the aorta. The patient was discharged in stable condition 20 days after successful repair of the aortic perforation.

**Discussion:**

Careful analysis of non-specific chest pain is crucial. This case highlights the importance of considering rare sequelae after multiple cardiac procedures, such as PPM and PCI. This case demonstrates the risk of aortic injury from extra-support guides during PCI, particularly near right atrial pacing leads.

Learning points

- Evaluate atypical chest pain for both ischaemic and non-ischaemic causes.
- Access the risk of potential sequelae in patients undergoing multiple cardiac procedures, including permanent pacemaker implantation and percutaneous coronary intervention (PCI).
- Recognize and mitigate the risk of aortic injury when using extra-support guide catheters during PCI, particularly near right atrial pacing leads.

## Introduction

In contemporary clinical practice, numerous patients undergo both percutaneous coronary intervention (PCI) and permanent pacemaker (PPM) implantation for the management of ischaemic heart disease and conduction disturbances. While PPM lead perforation is a rare complication, it typically occurs shortly after implantation. However, in our case, perforation was observed at an unprecedented 39 days post-implantation, notably just two days following PCI.

## Summary figure

**Figure:**
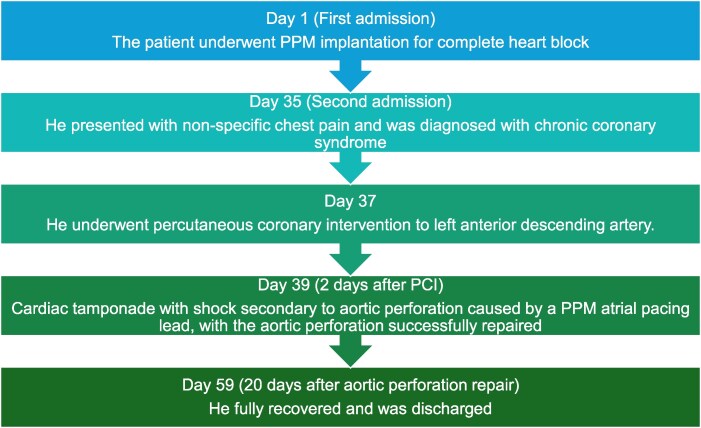


## Case presentation

**Figure 1:**
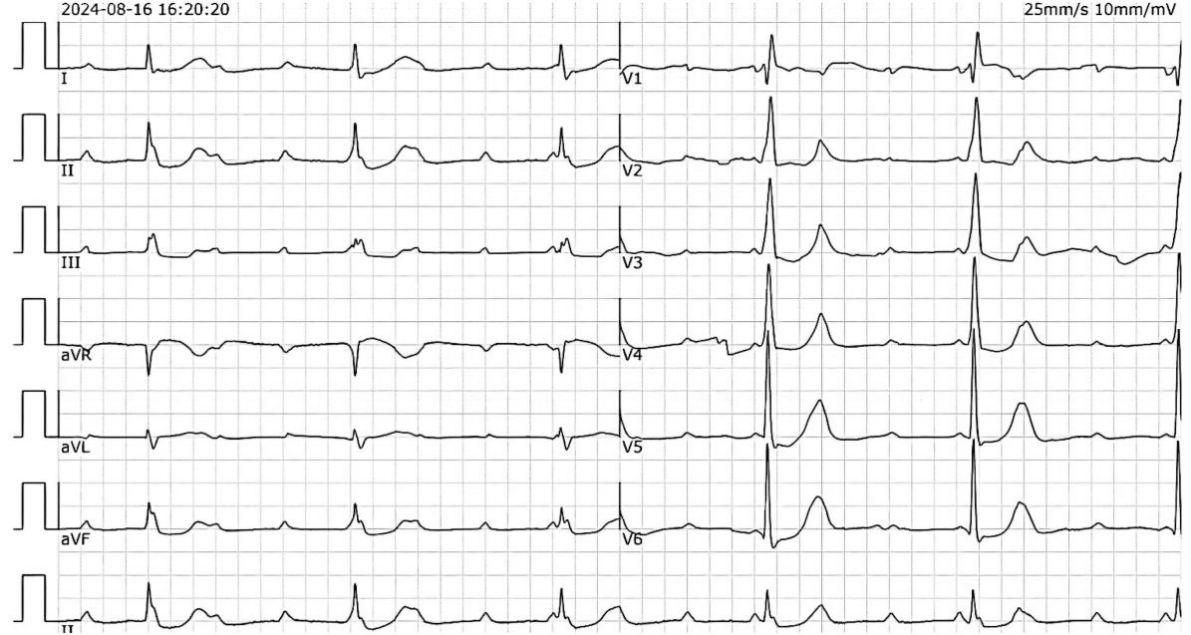
Electrocardiogram of the patient demonstrating complete heart block, which necessitated the implantation of a dual-chamber pacemaker during the initial admission.

**Figure 2:**
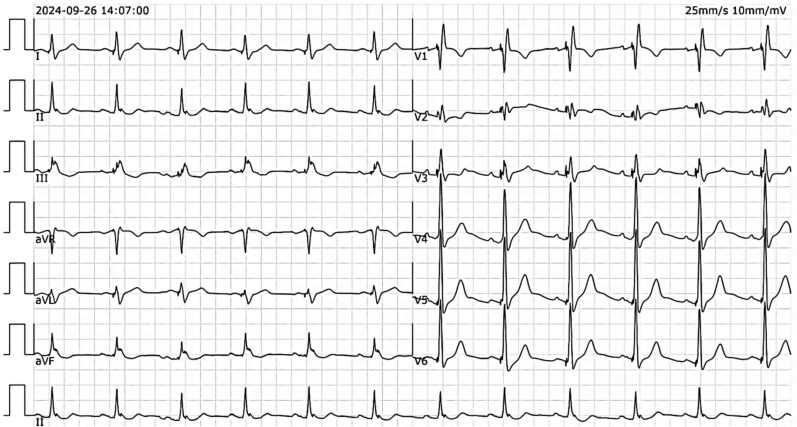
Electrocardiogram upon second admission, demonstrating normal sinus rhythm with right bundle brunch block, which showed no significant changes compared to previous post-pacemaker implantation ECG.

A 70-year-old Chinese man with a medical history significant for hypertension, diabetes, and complete heart block (status post-pacemaker insertion 6 weeks prior, *Figure 1*) presented with a 3-week history of intermittent, mild chest discomfort. These episodes were brief, lasting only a few minutes, and alleviated with rest. Physical examination was unremarkable, and his body mass index was within normal limits. Laboratory studies, including cardiac enzymes and routine chemistries, were all within normal limits, except for dyslipidaemia. An ECG demonstrated sinus rhythm with right bundle branch block (*Figure 2*). Echocardiography revealed septal dyskinesia, a normal ejection fraction of 66%, normal cardiac chamber dimensions, mild aortic regurgitation, and mild aortic root dilation measuring 44 mm.

**Figure 3:**
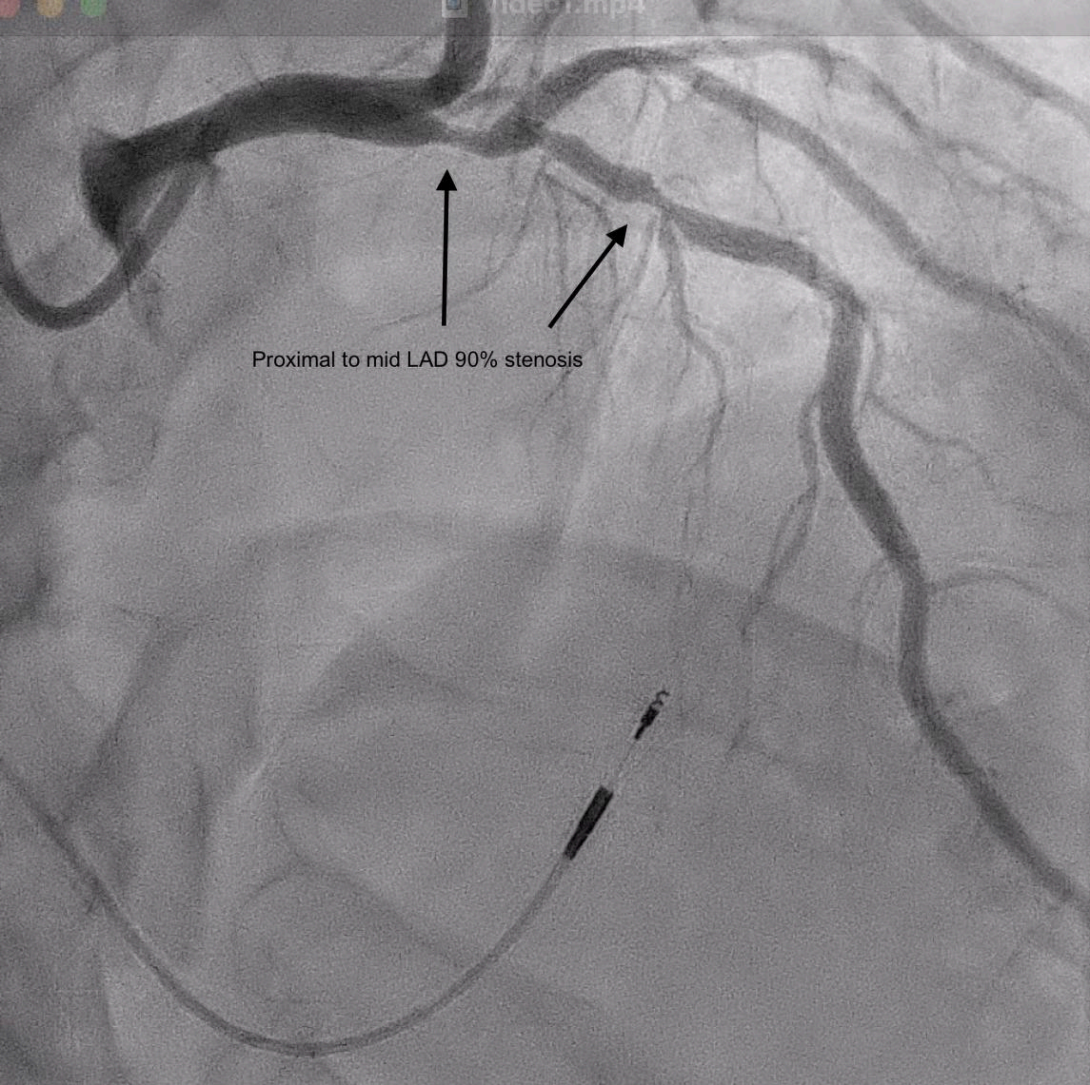
Diagnostic angiography (cine, right anterior oblique view) of the left coronary system demonstrating diffuse stenosis of the proximal to mid-segment of the left anterior descending artery, with up to 90% luminal narrowing.

Given his high cardiovascular risk profile, the patient was diagnosed with chronic coronary syndrome and underwent an invasive coronary angiogram. The procedure was performed using a J-Tip guidewire (Cordis, Cordis Corporation, Miami Lakes, FL, USA) and a 5 French TIG catheter (Terumo, Terumo Corporation, Tokyo, Japan) for diagnostic angiography. Angiographic findings revealed up to 90% stenosis in the proximal to mid-segment of the left anterior descending artery (LAD), while both the circumflex artery and right coronary artery appeared normal. Due to the significant LAD stenosis (*Figure 3*; Supplementary material online, *Video S1*), no physiological assessment was conducted for the lesion. For PCI, a 6 French Launcher EBU 3.5 (Extra Backup) guide catheter (Medtronic, Medtronic Inc., Minneapolis, MN, USA) was utilized. Two Runthrough NS floppy guidewires (Terumo, Terumo Corporation, Tokyo, Japan) were placed in the LAD and diagonal branch for lesion access and protection. Following lesion preparation, the proximal to mid-LAD was treated with a Promus PREMIER™ drug-eluting stent (3.5 × 32 mm) (Boston Scientific, Marlborough, MA, USA). Post-dilation and optimization were performed using non-compliance balloons; NC Sprinter (3.5 × 15 mm) (Medtronic, Medtronic Inc., Minneapolis, MN, USA) and Quantum (4.0 × 8 mm) (Boston Scientific, Marlborough, MA, USA). The final thrombolysis in myocardial infarction flow was grade 3, with no residual stenosis.

Two days after PCI, the patient developed dizziness and profuse sweating. His vital signs were notable for hypotension (67/45 mmHg), tachycardia (heart rate: 123/min), tachypnea (respiratory rate: 22/min), and an oxygen saturation of 90%. Bedside echocardiography revealed a large pericardial effusion with diastolic collapse of the right ventricular free wall. An emergency subcostal pericardiocentesis with autologous blood transfusion was performed, yielding a significant volume of bright red blood that rapidly re-accumulated. The catheterization lab was activated for a repeat angiogram to evaluate for PCI-related coronary perforation; however, no perforation was identified.

Upon reviewing the pacemaker implantation procedure performed 6 weeks earlier, a right subclavian vein puncture approach was utilized due to occlusion of the left brachiocephalic vein to the superior vena cava. A His bundle electrogram was not recorded. A Medtronic SelectSecure 3830 model lead (69 cm, 4.1 F, fixed helix length 1.8 mm) was placed using the double-wire technique, targeting the left bundle branch region. Pacing parameters included a threshold of 0.5 V at 0.5 ms, R-wave sensing of 7.0 mV, and an impedance of 657 ohms. Due to the steep axial angle of the right subclavian vein relative to the superior vena cava, positioning a Medtronic SelectSecure 3830 atrial lead (69 cm, 4.1 F, fixed helix length 1.8 mm) within the right atrial appendage (RAA) proved challenging. After multiple attempts, successful fixation was achieved using the screw-in method. The bipolar threshold measured 0.9 V at 1.0 ms, with *P*-wave sensing at 2.3 mV and impedance of 452 ohms. The lead was connected to a MRI-compatible DDDR pulse generator (A3DR01) (Medtronic, Medtronic Inc., Minneapolis, MN, USA). While no post-procedure chest X-ray was performed, fluoroscopic recording was available for review (see Supplementary material online, *Video S2 and S3*).

**Figure 4:**
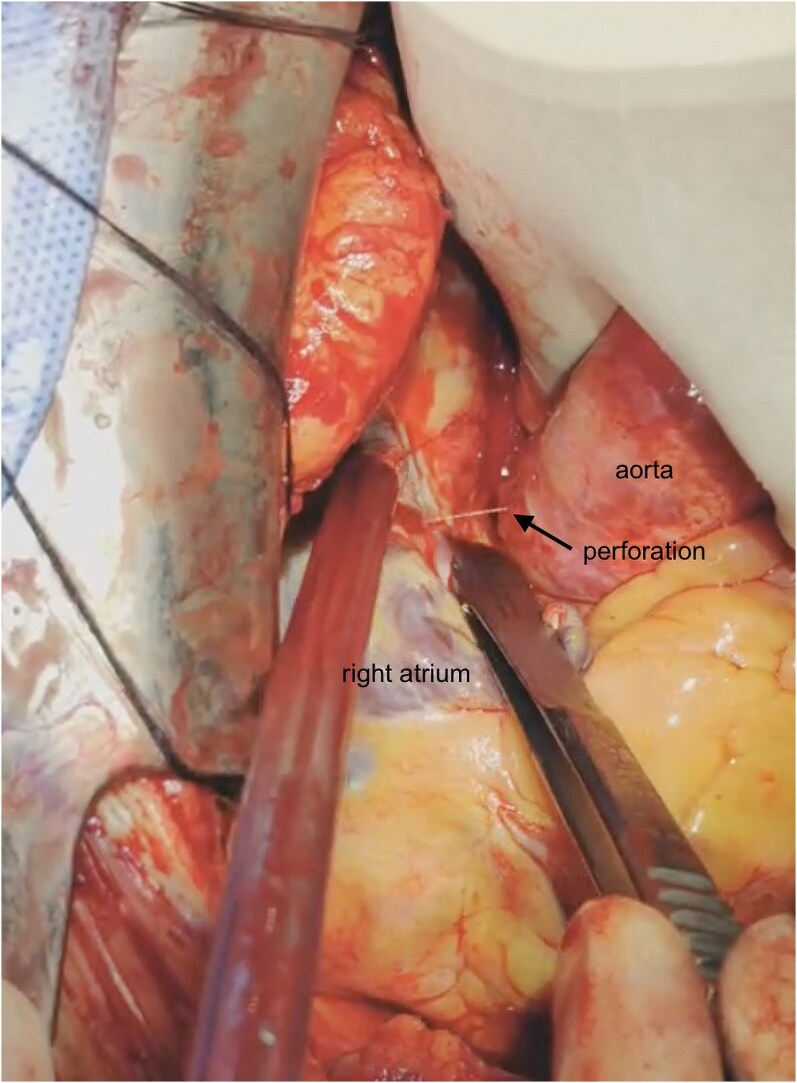
Intra-operative photograph from an open thoracotomy, demonstrating a 2 mm puncture on the anterolateral ascending aortic wall, just above the sinus of Valsalva, near the right atrium.

Suspecting lead perforation, an emergency open thoracotomy was performed. Pre-operative evaluation of pacing parameters remained unchanged. Intra-operative findings revealed high pericardial cavity pressure with a large volume of fresh blood and clots. A 2-mm rupture was identified on the anterolateral wall of the ascending aorta, just above the sinus of Valsalva, with active bleeding during each cardiac contraction (*Figure 4*; Supplementary material online, *Video S4*). This rupture was repaired using double-armed 4–0 Prolene sutures with pledgets. Additionally, the pacing lead tip was noted to be exposed at the RAA. To reinforce the site, a 5–0 cardiovascular Prolene cushion suture was applied. Repositioning of the pacing lead was not required.

During the hospital stay, the patient developed a chest infection, which was managed with broad-spectrum antibiotics. The patient’s pacing parameters remained stable throughout the hospital course. He made a full recovery and was discharged 20 days after the perforation.

## Discussion

Symptoms related to pacemaker lead perforation vary widely, ranging from asymptomatic (14%), often identified by abnormal lead parameters, to symptomatic cases (86%), which most commonly present as non-cardiac chest pain (46%).^1^ Initially, we considered chronic coronary syndrome in our patient; however, his atypical chest pain may have been due to irritation caused by the pacing lead.

**Figure 5:**
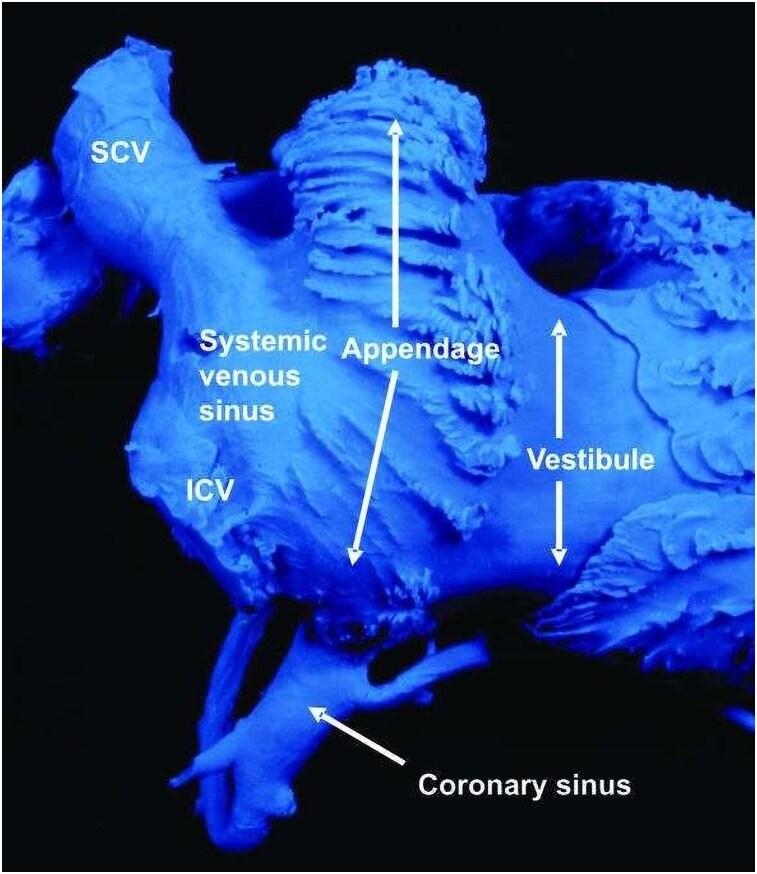
A cast of the right atrium, photographed in lateral projection from the right side, shows how the pectinate appendage interposes between the systemic venous sinus [receiving the superior and inferior caval veins (SCV and ICV) and the coronary sinus] and the vestibule of the tricuspid valve. [This figure has been adapted from Reference 3 (*Figure 16*)].

The right atrium composed of a venous component, an appendage, a vestibule, body and the septum, which separates it from the left atrium. The RAA is roughly triangular, with ridges representing the pectinate muscles, and its apex pointing upwards. It forms the entire anterior wall of the right atrium and overlaps the aortic root^2,3^ (*Figure 5*). In this case, the challenging anatomy of the right subclavian vein complicated pacemaker lead delivery to the right atrium. Due to its thinner wall compared to the right ventricle, the right atrium is particularly susceptible to perforation, especially when a pacing lead is placed in the RAA under excessive pressure.^4^ Although the RAA is a generally safe pacing site, multiple attempts at lead placement and the use of a screw-in active fixation mechanism may have contributed to myocardial injury, inflammation, and eventual perforation.^4–6^

A CT scan conducted during the patient’s second admission, 2 days before PCI, demonstrated an intact pericardium. The right atrial pacing lead was positioned in the RAA, though it was in close proximity to and oriented towards the ascending aorta (see Supplementary material online, *Figure S1A*–*D*). We used an EBU passive-support guide catheter, which enhances backup support due to its stiff shaft and elongated secondary curve that rests against the contralateral aortic wall.^7^ This strong backup support likely exerted significant pressure on both the aortic wall and the adjacent right atrium, potentially injuring the myocardium and increasing the risk of pacing lead perforation into the aorta (see Supplementary material online, *Figure S2*). Contact between the guiding catheter and aortic wall near the pacemaker lead was likely to occur during engagement of the left main ostium or during insertion of the long stent (3.5 mm × 32 mm). However, this contact did not persist throughout the entire PCI procedure. After engagement, DSA imaging focused solely on the guide tip, wire, and lesions during PCI (see Supplementary material online, *Video S5*).

Although antiplatelet therapy is well known to increase bleeding risk, its direct link to cardiac perforation remains uncertain.^4^ However, one study has suggested a potential association between antiplatelet use and cardiac perforation.^8^ Our patient was initially prescribed aspirin (100 mg) daily. Upon his second admission, he received a loading dose of dual antiplatelet therapy (aspirin 300 mg and clopidogrel 300 mg), followed by maintenance dual antiplatelet therapy (aspirin 100 mg and clopidogrel 75 mg). During PCI, he was administered unfractionated heparin (6500 units; 100 units/kg) based on body weight. This anticoagulation regimen may have exacerbated bleeding from a previously injured aorta. Although some studies have linked steroid use to cardiac perforation,^9^ it was not a contributing factor in this case.

## Conclusion

While the exact cause of the aortic perforation remains unclear, multiple factors likely contributed. To mitigate the risk, alternative pacing site, such as the inter-atrial septum, could be considered, though this approach has its own limitations.^6^ For future PCI procedures, utilizing guide catheters with reduced support, such as Judkins catheters, may be advantageous. Additionally, adopting a trans-femoral approach instead of a trans-radial one could be beneficial, as it provides ∼1.6 times less backup support due to the guide catheter’s more horizontal trajectory in the aorta and its lower contact point with the opposite aortic wall.^7^

## Lead author biography


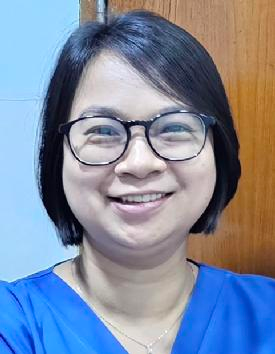


Dr. Toe Wai Wai Naing earned her medical degree from the University of Medicine (1), Myanmar, and has obtained prestigious qualifications, including Membership of the Royal College of Physicians (MRCP, UK) and Fellowship of the Royal College of Physicians (FRCP, Edinburgh). She is also recognized as a Fellow of the ASEAN College of Cardiology. Currently, she is pursuing an Interventional Cardiology Fellowship at Xiamen Cardiovascular Hospital, affiliated with Xiamen University. Outside of her professional endeavours, she enjoys growing cacti as a hobby.

## Supplementary Material

ytaf298_Supplementary_Data

## Data Availability

Data underlying this case report are available in the article and supplementary materials. Additional clinical data are not publicly available due to patient privacy but may be obtained from the corresponding author upon reasonable request, subject to institutional and ethical approvals.
