# Supplementary figures and images for: Aortic perforation following percutaneous coronary intervention in a patient with permanent pacemaker: a case report
Source: Eur Heart J Case Rep. 2025 Jun 24;9(7):ytaf298. doi: 10.1093/ehjcr/ytaf298 (PMC12243536; doi:10.1093/ehjcr/ytaf298)

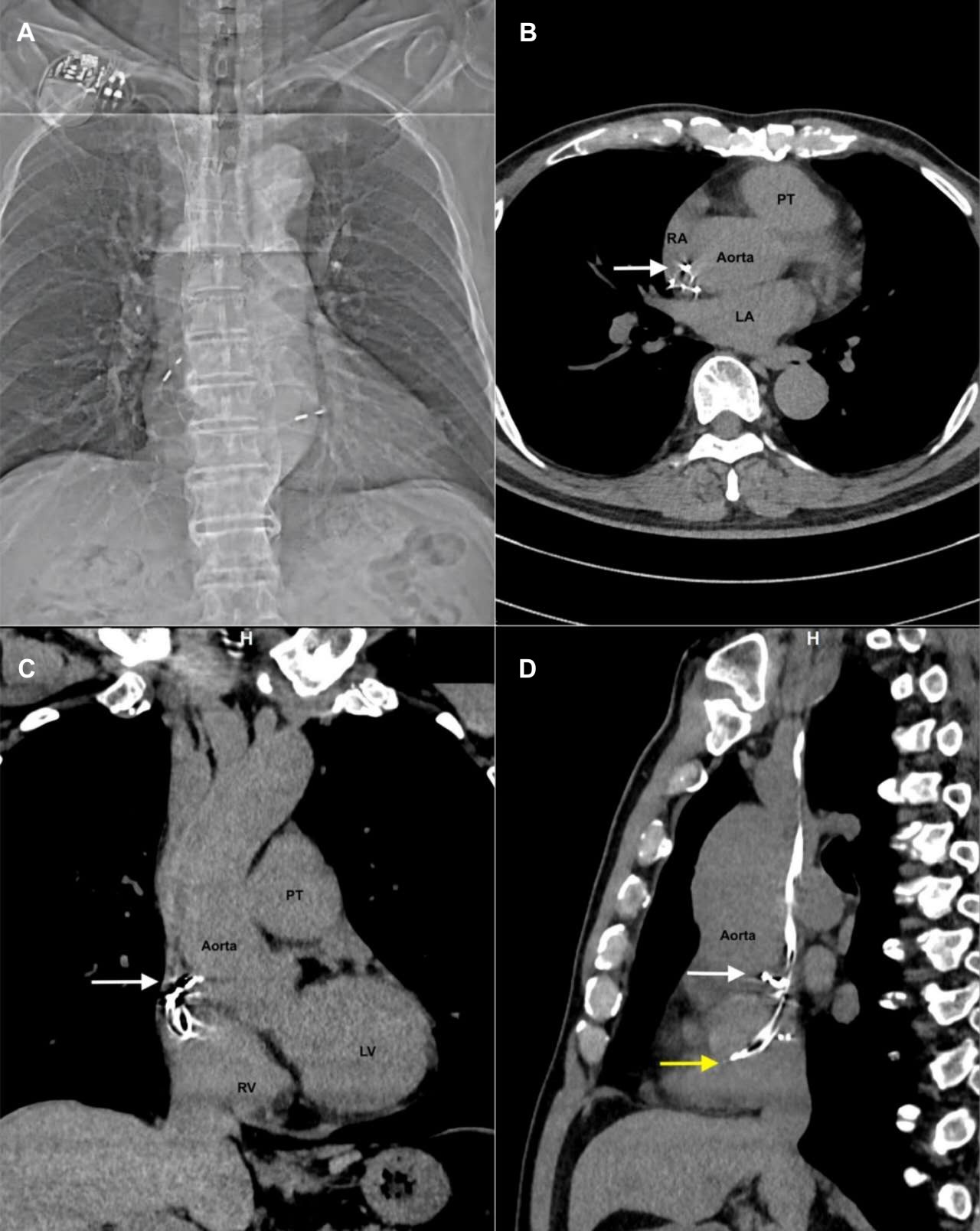

Supplement: ytaf298_Supplementary_Data [file ytaf298_supplementary_data.zip › FigureS1.jpeg]

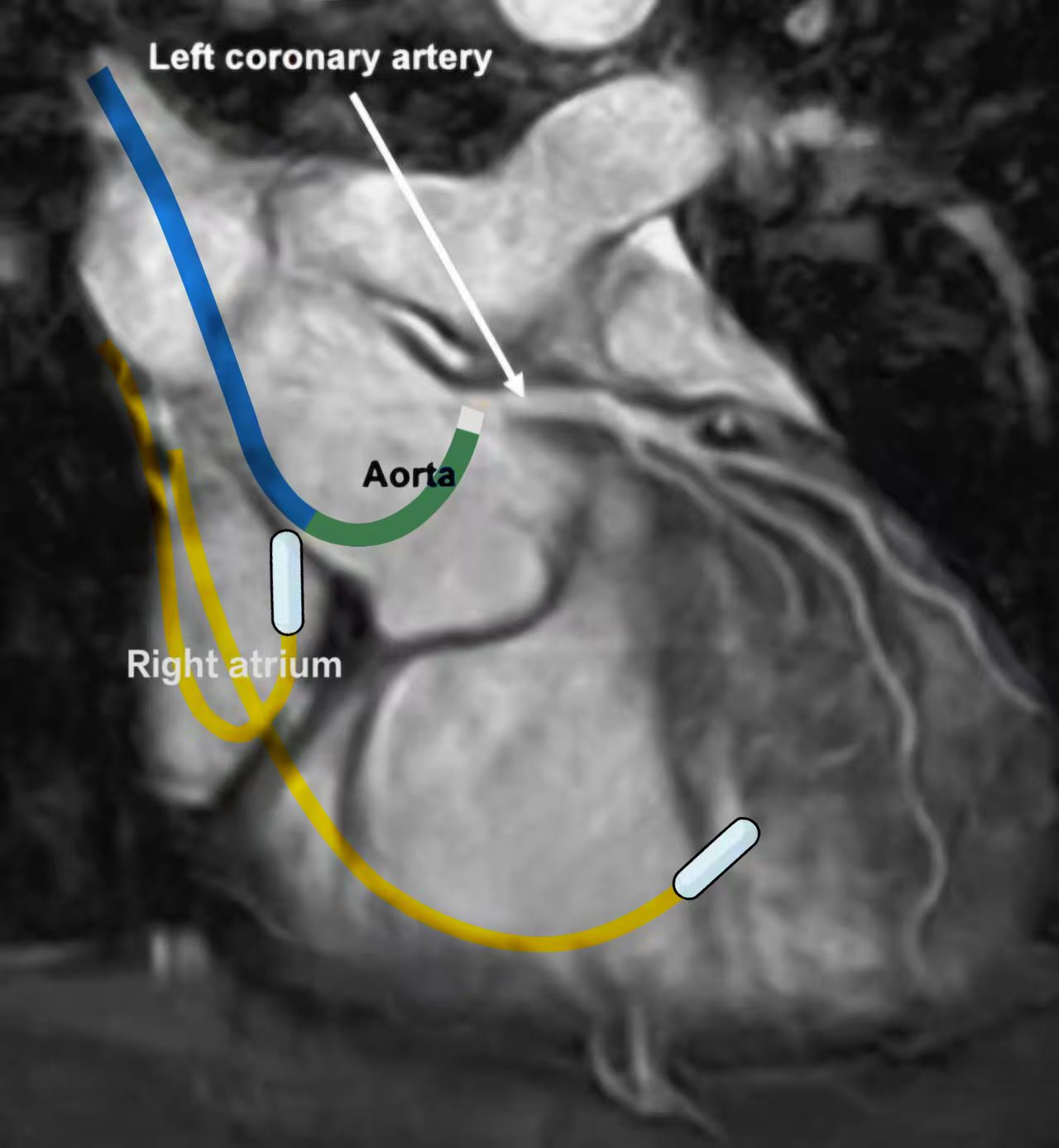

Supplement: ytaf298_Supplementary_Data [file ytaf298_supplementary_data.zip › FigureS2.jpg]
